# Pruritus Features in Children with End-Stage Renal Disease Underwent Dialysis: A Cross-Sectional Study

**DOI:** 10.1155/2021/9970321

**Published:** 2021-07-28

**Authors:** Mehryar Mehrkash, Seyed-javad Golestaneh, Yahya Madihi, Fatemeh Paknazar, Mahdi Hadian, Mojtaba Akbari, Bahareh Abtahi-Naeini

**Affiliations:** ^1^Imam Hossein Children's Hospital and Child Growth and Development Research Center, Research Institute for Primordial Prevention of Non Communicable Disease, Isfahan University of Medical Sciences, Isfahan, Iran; ^2^School of Medicine, Isfahan University of Medical Sciences, Isfahan, Iran; ^3^Social Determinants of Health Research Center, Semnan University of Medical Sciences, Semnan, Iran; ^4^Department of Epidemiology, School of Health, Isfahan University of Medical Sciences, Isfahan, Iran; ^5^Pediatric Dermatology Division of Department of Pediatrics, Imam Hossein Children's Hospital, Isfahan University of Medical Sciences, Isfahan, Iran; ^6^Skin Diseases and Leishmaniasis Research Center, Isfahan University of Medical Sciences, Isfahan, Iran

## Abstract

**Objective:**

Evaluation of the pruritus features in children with end-stage renal disease (ESRD) who underwent dialysis at an academic tertiary pediatric dialysis center.

**Methods:**

This cross-sectional study was conducted at an academic tertiary pediatric dialysis center, Isfahan, Iran. The reviewed medical records of the children included their characteristics, dialysis properties, and laboratory parameters. The 4-item itch questionnaire was utilized to assess distribution, severity, frequency, and associated sleeping disorders.

**Results:**

Thirty ESRD patients with pruritus, including 23 males (76.7%) with a mean age of 11.7 ± 3.64 years, were recruited. The most common cause of CKD was nephronophthisis (23.3%). The median total score of pruritus was 5 (range: 3-15). The distribution score of pruritus was directly correlated with the age (Spearman's rho = 0.42, *P* = 0.02) and serum level of parathyroid hormone (PTH) (Spearman's rho = 0.42, *P* = 0.04). In the reduced multiple logistic regression model, the increasing level of serum calcium was associated with increased odds of having total pruritus score ≥ 5 (OR (odds ratio): 4.5; 95% CI 1.12 to 18.05). In addition, an increase in age for one year was found to be associated with 50% higher odds of having total pruritus score ≥ 5 (OR: 1.5; 95% CI 1.03 to 2.18).

**Conclusion:**

Increased level of serum Ca and higher age were associated with increased odds of having more severe pruritus score in children.

## 1. Introduction

Chronic kidney disease (CKD) and end-stage renal disease (ESRD) and its complications are a growing problem worldwide in children [1, 2]. Regardless of CKD cause, it may be accompanied with various skin lesions, including pruritus [3–5]. Pruritus, as a common and distressing symptom among patients with CKD, poses a high burden, and decreases quality of patients' life [6, 7]. Etiology of uremic pruritus may comprise several factors, including xerosis, peripheral neuropathy, mast cell hyperplasia, increased serum level of histamine, vitamin A, parathyroid hormone (PTH), and certain inflammatory factors, such as interleukin-2 (IL2) [8, 9].

Finding an approach to the treatment of children with pruritus is believed to be a challenging issue for two major reasons; primarily, there are not many studies concerning itching and associated factors in children and adolescents with ESRD; secondly, the exact etiology and pathophysiology of CKD-associated pruritus remains unclear and is uncommon in children [10].

Knowledge about the characteristics and related factors to CKD-associated pruritus is important for the identification of possible risk factors, implementing effective and appropriate intervention for improving the clinical manifestation, and allocating enough health care resources.

Certain studies have been conducted to evaluate the clinical characteristics and etiology of uremic pruritus in adult patients [11–13]; however, there are a limited number of studies with epidemiological information on ESRD-associated pruritus in children [3, 14]. The scarcity of epidemiological information is even more pronounced in developing countries. Thus, it is necessary to conduct further studies on the role of clinicoepidemiogical and socioeconomic factors in the management of uremic pruritus. Therefore, the current study is aimed at providing itching characteristics and exploring its association with the selected related factors in ESRD-associated pruritus in children under dialysis.

## 2. Material and Methods

### 2.1. Study Population

This cross-sectional study was conducted at an academic tertiary pediatric dialysis center in Imam Hossein Children's Hospital, affiliated to Isfahan University of Medical Sciences. The project was approved by the Ethical Committee of Isfahan University of Medical Sciences (IR.MUI.MED.REC.1399.571), in agreement with the last version of Helsinki Declaration. Written informed consent for participation was obtained from all the children and their parents/guardian after the objective, and the procedure of our work were completely explained.

The inclusion criteria for case selection were (i) definite diagnosis of ESRD, (ii) initiation of dialysis at least 6 months beforehand, (iii) age at the onset of CKD below 18 years, and (iv) children with 3-18 years old. The exclusion criteria were changes in the mental status making the patient unable to make a detailed assessment of itch, known case of severe liver disease, and history of chronic dermatologic diseases, such as atopic dermatitis, psoriasis, and scabies. Additionally, those who refused to participate were excluded.

### 2.2. Data Collection

The reviewed medical records of the children included their sex, age, causes of ESRD, dialysis type and duration, complications related to dialysis, age at onset of dialysis, urinary output status, comorbidity, and history of consanguineous marriage of parents. The laboratory tests were performed according to a protocol of routine blood tests for the dialyzed patients and were measured with routine methods in our center. Serum blood urea nitrogen (BUN), creatinine (Cr), calcium (Ca), phosphorus (P), Ca×P product, and parathyroid hormone (PTH) level were determined in all the participants. These parameters were assessed over the previous 6 months and the mean, minimum, and maximum of each parameter were noted.

### 2.3. Pruritus Assessment

The 4-item itch questionnaire (4IIQ) was utilized to assess distribution, severity, frequency, and the associated sleeping disorders [1]. The questionnaire estimated the extent of pruritus (1–3 points), intensity (1–5 points), frequency (1–5 points), and sleep disturbances (0–6 points) caused by itching during the seven days prior to the examination. The ratings ranged from 3 (mild pruritus) to 19 points (very severe itching) [2, 3].

### 2.4. Statistical Analysis

Numerical variables were described with the mean, standard deviation (SD), median, and range (minimum and maximum) of the values. Frequency distribution tables were used to report the count and percentage of categorical variables. Based on the total pruritus score of 5, the patients were divided into two groups with lower (≤5) and higher (>5) severity. The analysis was performed through two stages, namely, the preliminary stage to select the most important variables, and the main stage for modeling the selected variables. The marginal relationship between each categorical variable and the two groups was initially evaluated via Chi-square or Fisher's exact tests; the rest of the variables (numerical ones) were compared between the two groups employing *t*-test or Mann-Whitney *U* test regarding the normal distribution assumption. The normal distribution assumption was checked with the Shapiro-Wilk test. The associations between the numerical variables and pruritus scores were also assessed with Spearman's rho correlation coefficients. The variables that were significantly (*P* < 0.05) or liberally (*P* < 0.2) related to the pruritus score in the first stage entered the final analysis. In this stage, to examine the relationship between each of the explanatory variables and being in the group with a higher score as the dependent variable, simple and multiple logistic regression models were fitted on the data and the final reduced model was obtained via stepwise (backward LR) method and was interpreted. Statistical analysis was performed with SPSS-18 software at 95% confidence level for the statistical tests.

## 3. Results

### 3.1. Demographic and Laboratory Data/Study Population

Thirty CKD children with pruritus were recruited. The mean age of the patients was 11.7 ± 3.64 (range 3-18) years, and there were 23 males (76.7%). The most frequent causes of CKD were nephronophthisis (23.3%), hypoplastic kidney (10%), and FSGS (10%). MBD, anemia, and HTN were noted in 93.3%, 90%, and 80% of the patients, respectively. The mean dialysis duration was 34 ± 23.4 (range 9-108) months.  Table 1 represents the demographics and laboratory parameters of the patients enrolled in this study.

### 3.2. Pruritus Features

The mean total score of pruritus was 5.37 ± 2.41. The median total score was 5 (range: 3-15). Seventeen patients (56.7%) had total score ≥ 5. Frequency of pruritus features included distribution, severity, episodes, and sleep disturbances in the patients, which are shown in Table 2. Dry skin was observed in all the subjects. Most patients had multiple location distributions (18/30), itching with the need to scratch, but without excoriation (14/30), four short episodes or one long episode (29/30), and no episode of awakening due to pruritus (27/30) (Table 2).

### 3.3. Characteristics of the Patients according to Total Pruritus Score

Tables 3 and 4 depict the distribution of general characteristics as well as the laboratory parameters and continuous variables according to the total pruritus score. Distribution of sex, dialysis type, dialysis-related complications, urinary outputs, and consanguineous marriage of parents were not significantly different between total pruritus score < 5 and ≥5 (*P* > 0.05) (Table 3). The subjects with higher serum level of Ca had higher total pruritus score ≥ 5 (9.41 ± 0.83 in total pruritus score ≥ 5 versus 8.76 ± 0.92 in total pruritus score < 5; *P* = 0.05) (Table 4).

### 3.4. Correlation between Quantitative Variable and Pruritus Score

Correlation analysis was performed between the quantitative characteristic and pruritus score (Table 5). The distribution score of pruritus was directly correlated with the age of the patients (Spearman's rho = 0.42, *P* = 0.02) and the serum level of PTH (Spearman's rho = 0.42, *P* = 0.04). There were no significant correlations between the other laboratory parameters and pruritus score (*P* > 0.05 for all correlations) (Table 5).

### 3.5. Logistic Regression Model for Association between Pruritus and Explanatory Variables

Table 6 demonstrates the results of simple, multiple, and reduced multiple logistic regression models concerning the association between total pruritus score and explanatory variables. In the reduced multiple logistic regression model, the increase in the level of serum Ca was associated with the increased odds of having total pruritus score ≥ 5 (OR (odds ratio): 4.5; 95% CI 1.12 to 18.05). In addition, an increase in age by one year was associated with 50% higher odds of having total pruritus score ≥ 5 (OR: 1.5; 95% CI 1.03 to 2.18) (Table 6).

## 4. Discussion

Our study revealed that most patients had mild severity of pruritus. The results demonstrated that the increase in the level of serum Ca and patients' age were associated with increased odds of having more severe pruritus. These findings could provide new insights into the clinical and epidemiological aspects of pruritus associated with ESRD in children.

In line with the study by Schwab et al., most of the children in our study had mild pruritus severity [10].

In this paper, the increase in age was found to be related to the increased severity of pruritus. This relation between age and severity could be attributed to the CD4 T cell differentiation into the Th1 cell along with the increase in age. Increased Th1 will produce greater IL-2, which is an important mediator in uremic itching [10].

In our study, all the patients had dry skin, possibly due to disturbed skin barrier that is intensified as the disease progresses and when the CKD progresses to ESRD; it seems logical in the presence of pruritus in most patients [3, 15]. Moreover, none of our children used any kinds of moisturizers during the two months before. Meanwhile, study in adults demonstrated that in patients undergoing hemodialysis, the use of emollients significantly improved dry skin [16].

Several studies have suggested that skin dryness in CKD is an important factor for pruritus associated with CKD [17, 18]. Children with ESRD undergoing dialysis reported skin dryness more often than those receiving conservative treatment [3]. Disturbed skin barrier in skin dryness may either facilitate or aggravate the development or intensity of pruritus. The high prevalence of dry skin in our study may be associated with the selection criteria in which all the patients had ESRD compared to studies with multistage CKD.

In the present research, there were no significant differences between the type of hemodialysis and severity of itching. On the one hand, children undergoing peritoneal dialysis had less skin moisture than those undergoing hemodialysis and healthy controls and there was a significant correlation between lower moisturizing of the stratum corneum and itching [19]. On the other hand, skin dryness may be influenced by race, accompanying illnesses, and various environmental factors [20]. Therefore, it seems as though the relation between the type of dialysis and the related factors with severity of pruritus is of great importance and should be addressed in the future.

The exact role of divalent ions, as the cause of or as a related factor to severity uremic pruritus, has been a controversial issue. Herein, the mean serum level of Ca during the previous 6 months was associated with intensity of pruritus. The effect of divalent ions on pruritus in children with ESRD is attributed to the nerve modulation and histamine release by mast cells [21]. Nonetheless, the role of histamine release by mast cells in ESRD-associated pruritus is uncertain. Thus, the effect of divalent ions, nerve modulation, and histamine release by mast cells, as a possible mechanism for uremic pruritus, still needs further investigation [22, 23].

Disappearance of uremic pruritus following parathyroidectomy was observed herein although this was a temporary event [10]. This finding is consistent with that of Harlim, reporting no significant correlations between the increased PTH and uremic pruritus [24]. Similarly, in our study, the increase in the PTH level was not correlated with the intensity of pruritus yet was interestingly associated with distribution of pruritus.

Our work had certain limitations, including the small sample size. Although the number of children with pruritus associated with ESRD was not large compared to the adult, it seemed to be an important factor for spotting significant differences between pruritus and the related factors. Lack of laboratory measurement exactly at the time of evaluation for pruritus was another limitation, which is also present in other similar studies. Furthermore, lack of some factors that were not evaluated in our study, such as plasma histamine level, cutaneous mast cell proliferation, opioid peptides, aluminum level, and inflammatory markers, could be considered another limitation; this might be important in children due to the problem in evaluation facilities.

Despite these limitations, our study investigated detailed characteristics of children with ESRD and applied a comprehensive scale to characterize itching in them. The present work could also provide references for the characteristics of pruritus in these children, about which there is limited data.

## 5. Conclusion

The obtained results herein suggested that pruritus associated with ESRD had a low score of severity among most children. Furthermore, the increase in the level of serum Ca and higher age could be associated with increased odds of having more severe pruritus in children. In the future, large-scale studies should be designed on pediatric populations to improve our understanding of this spectrum of disorders in children.

## Figures and Tables

**Table 1:** Clinical characteristics and laboratory data of chronic kidney disease (CKD) children with pruritus.

Sex		
Male (*n*, %)	23	76.7
Female (*n*, %)	7	23.3
Dialysis type		
Peritoneal (*n*, %)	11	36.7
Hemodialysis (*n*, %)	19	63.3
Complication of dialysis		
Anemia (*n*, %)	27	90.0
MBD (*n*, %)	28	93.3
HTN (*n*, %)	24	80.0
Acidosis (*n*, %)	18	60.0
Lipid disorder (*n*, %)	5	16.7
Thyroid disease (*n*, %)	4	13.3
Urinary output		
Complete anuria (*n*, %)	7	23.3
Relative anuria (*n*, %)	13	43.3
Normal (*n*, %)	6	20.0
Polyuria (*n*, %)	4	13.3
Comorbidity		
No (*n*, %)	21	70.0
Yes (*n*, %)	9	30.0
Consanguineous marriage		
Yes (*n*, %)	23	76.7
No (*n*, %)	7	23.3
Age (year)		
Mean (SD)	11.7	(3.64)
Median (min-max)	13	3-18
Dialysis duration (month)		
Mean (SD)	34	(23.4)
Median (min-max)	24	9-108
Age at onset of dialysis (year)		
Mean (SD)	9.09	(4.15)
Median (min-max)	8.5	0-15
Serum BUN (mg/dl)		
Mean (SD)	53.27	(24.19)
Median (min-max)	49.5	24-124
Serum Cr (mg/dl)		
Mean (SD)	6.82	(2.57)
Median (min-max)	6.6	1.35–11.3
Serum Ca (mg/dl)		
Mean (SD)	9.13	(0.92)
Median (min-max)	9.2	7.2-11.4
Serum P (mg/dl)		
Mean (SD)	5.43	(1.49)
Median (min-max)	5.4	2.7–9.2
Serum Ca×P		
Mean (SD)	49.7	(14.46)
Median (min-max)	47.7	26.2-75.4
Serum PTH (ng/ml)		
Mean (SD)	524.12	(313.53)
Median (min-max)	594	92-1079

MBD: mineral bone disorder; HTN: hypertension; BUN: blood urea nitrogen; Cr: creatinine; Ca: calcium; P: phosphorus; PTH: parathyroid hormone.

**Table 2:** Pruritus features of patients enrolled in the study.

Pruritus feature	Categories	Count	%
Distribution	Single location	11	36.7
	Multiple locations	18	60.0
	Generalized	1	3.3
Severity	Without the need to scratch	6	20.0
	With the need to scratch but without excoriation	14	46.7
	Unrelieved by scratching but without excoriation	5	16.7
	Accompanied by excoriation	4	13.3
	Totally restless	1	3.3
Frequency	Four short episodes or one long episode	29	96.7
	Continuous	1	3.3
Sleep disturbances	One episode of awakening due to pruritus	2	6.7
	Two episodes of awakening due to pruritus	1	3.3

**Table 3:** Distribution of general characteristics of chronic kidney disease (CKD) children with pruritus according to the total pruritus score.

Characteristics		Pruritus				*P* value
		Total score < 5		Total score ≥ 5		
		Count	%	Count	%	
Sex	Male	11	84.6	12	70.6	0.43
	Female	2	15.4	5	29.4	
Dialysis type	Peritoneal	5	38.5	6	35.3	0.99
	Hemodialysis	8	61.5	11	64.7	
Anemia	No	0	0	3	17.6	0.24
	Yes	13	100.0	14	82.4	
MBD	No	0	0	2	11.8	0.49
	Yes	13	100.0	15	88.2	
HTN	No	1	7.7	5	29.4	0.19
	Yes	12	92.3	12	70.6	
Acidosis	No	4	30.8	8	47.1	0.46
	Yes	9	69.2	9	52.9	
Lipid disorder	No	11	84.6	14	82.4	0.99
	Yes	2	15.4	3	17.6	
Thyroid disease	No	11	84.6	15	88.2	0.99
	Yes	2	15.4	2	11.8	
Comorbidity	No	8	61.5	13	76.5	0.44
	Yes	5	38.5	4	23.5	
Urinary output	Abnormal	10	76.9	14	82.4	0.99
	Normal	3	23.1	3	17.6	
Consanguineous marriage of parents	No	3	23.1	4	23.5	0.99
	Yes	10	76.9	13	76.5	

MBD: mineral bone disorder; HTN: hypertension.

**Table 4:** Laboratory parameters and continuous variables chronic kidney disease (CKD) children with pruritus according to the total pruritus score.

Variables		Pruritus										*P* value
		Total score < 5					Total score ≥ 5					
		Mean	SD	Med	Min	Max	Mean	SD	Med	Min	Max	
Age (year)		11	3.67	10	3	16	12.24	3.63	13	4	18	0.36
Duration of dialysis (months)		32.77	26.87	24	9	108	34.94	21.24	36	12	96	0.51
Age of onset of dialysis (year)		8.55	4.24	8	0	14	9.51	4.16	10	0	15	0.54
Serum BUN (mg/dl)	Mean	54.85	24.38	52	24	114	52.06	24.73	46	24	124	0.76
	Min	43.15	17.84	41	15	82	37.29	18.94	32	16	92	0.39
	Max	70.62	34.02	63	32	154	67	32.75	63	25	148	0.77
Serum Cr (mg/dl)	Mean	6.68	2.51	6.20	2.40	11.30	6.93	2.68	6.80	1.35	10.60	0.79
	Min	5.80	2.32	5.90	2.00	11.00	5.85	2.50	5.70	1.30	10.20	0.96
	Max	7.90	2.96	7.90	3.30	13.30	8.06	3.04	8.50	1.40	11.90	0.89
Serum Ca (mg/dl)	Mean	8.76	0.92	8.80	7.20	10.10	9.41	0.83	9.30	8.20	11.40	0.05
	Min	7.82	1.11	8.30	6.20	9.40	8.71	1.05	8.80	7.20	10.80	0.03
	Max	9.70	1.22	9.60	7.50	12.10	10.09	0.80	9.70	9.30	12.00	0.30
Serum P (mg/dl)	Mean	5.80	1.97	5.50	2.70	9.20	5.14	0.94	5.10	3.70	7.10	0.23
	Min	4.68	0.96	4.70	3.20	7.30	4.54	0.65	4.70	3.20	5.50	0.97
	Max	7.22	2.29	7.80	4.10	11.80	6.20	1.37	5.90	4.60	10.00	0.14
Serum PTH (ng/ml)	Mean	475.8	327.13	368	92	1079	562.07	309.26	660	140	1077	0.51
	Min	374.91	322.6	178	92	1030	498.9	294.05	536	122	1029	0.33
	Max	576.82	369.8	594	92	1128	625.3	340.4	710	140	1125	0.74
Serum Ca×P		50.87	17.93	47.85	26.19	75.44	48.80	11.66	47.50	30.34	68.40	0.71

BUN: blood urea nitrogen; Cr: creatinine; Ca: calcium; P: phosphorus; PTH: parathyroid hormone; Med: median; Min: minimum; Max: maximum.

**Table 5:** Correlation between quantitative variables and pruritus score in terms of Spearman's rho.

Variable		Correlation coefficient (*P* value)				
		Distribution score	Severity score	Frequency score	Sleep score	Total score
Age (year)		0.42 (0.02)	-0.12 (0.54)	0.01 (0.96)	0.02 (0.89)	0.17 (0.37)
Duration of dialysis (months)		0.21 (0.27)	-0.10 (0.61)	0.08 (0.69)	0.14 (0.47)	0.03 (0.87)
Age of onset of dialysis (year)		0.28 (0.13)	-0.13 (0.50)	0.05 (0.78)	0.04 (0.83)	0.09 (0.64)
Serum BUN (mg/dl)	Mean	0.08 (0.68)	0.13 (0.47)	0.22 (0.23)	0.06 (0.74)	0.03 (0.84)
	Min	-0.06 (0.76)	0.07 (0.70)	0.17 (0.36)	0.12 (0.50)	-0.05 (0.79)
	Max	0.13 (0.47)	0.13 (0.48)	0.19 (0.31)	0.01 (0.92)	0.04 (0.82)
Serum Cr (mg/dl)	Mean	0.12 (0.51)	-0.11 (0.57)	0.01 (0.95)	0.08 (0.65)	0.02 (0.91)
	Min	0.11 (0.57)	-0.15 (0.42)	-0.01 (0.95)	0.05 (0.77)	-0.01 (0.94)
	Max	0.10 (0.60)	-0.09 (0.61)	-0.05 (0.78)	0.09 (0.64)	0.02 (0.90)
Serum Ca (mg/dl)	Mean	0.15 (0.41)	0.18 (0.33)	-0.19 (0.31)	0.09 (0.62)	0.29 (0.12)
	Min	0.09 (0.63)	0.27 (0.15)	-0.14 (0.46)	0.06 (0.76)	0.34 (0.06)
	Max	0.24 (0.19)	0.03 (0.87)	-0.19 (0.30)	0.11 (0.57)	0.17 (0.36)
Serum P (mg/dl)	Mean	-0.05 (0.78)	-0.23 (0.22)	-0.25 (0.18)	-0.13 (0.51)	-0.23 (0.22)
	Min	0.23 (0.23)	-0.15 (0.44)	0.02 (0.90)	0.21 (0.26)	0.01 (0.94)
	Max	-0.01 (0.97)	-0.23 (0.21)	-0.15 (0.43)	-0.12 (0.52)	-0.24 (0.19)
Serum PTH (ng/ml)	Mean	0.33 (0.11)	-0.13 (0.54)	0.23 (0.28)	0.05 (0.80)	0.06 (0.79)
	Min	0.42 (0.04)	-0.12 (0.58)	0.20 (0.34)	0.06 (0.76)	0.12 (0.56)
	Max	0.14 (0.49)	-0.12 (0.57)	0.14 (0.50)	-0.07 (0.74)	-0.05 (0.80)
Serum Ca×P		0.04 (0.82)	-0.17 (0.36)	-0.22 (0.23)	-0.09 (0.64)	-0.11 (0.55)

BUN: blood urea nitrogen; Cr: creatinine; Ca: calcium; P: phosphorus; PTH: parathyroid hormone; Min: minimum; Max: maximum.

**Table 6:** Relationship of each of the most important study variables with the pruritus score based on logistic regression models.

Variables	*P* value	OR^1^	95% CI		*P* value	OR^2^	95% CI		*P* value	OR^3^	95% CI	
			Lower	Upper			Lower	Upper			Lower	Upper
Serum Ca (mean)	0.06	2.6	0.93	7.21	0.55	2.3	0.15	34.09	0.03	4.5	1.12	18.05
Serum Ca (min)	0.04	2.2	1.02	4.93	0.55	1.9	0.23	15.31	—	—	—	—
Serum P (max)	0.15	0.73	0.47	1.12	0.41	0.81	0.49	1.35	—	—	—	—
HTN	0.17	0.20	0.02	1.98	0.09	0.05	0.001	1.66	0.06	0.04	0.001	1.25
Age (year)	0.36	1.1	0.89	1.36	0.04	1.5	1.02	2.20	0.03	1.5	1.03	2.18

Ca: calcium; P: phosphorus; HTN: hypertension. ^1^Simple logistic regression models. ^2^Multiple logistic regression. model. ^3^Reduced multiple logistic regression model.

## Data Availability

Data available on request.
